# Serum Renalase Levels Are Predicted by Brain-Derived Neurotrophic Factor and Associated with Cardiovascular Events and Mortality after Percutaneous Coronary Intervention

**DOI:** 10.3390/jcm7110437

**Published:** 2018-11-12

**Authors:** I-Te Lee, Wayne Huey-Herng Sheu

**Affiliations:** 1 Division of Endocrinology and Metabolism, Department of Internal Medicine, Taichung Veterans General Hospital, Taichung 40705, Taiwan; whhsheu@vghtc.gov.tw; 2 School of Medicine, National Yang-Ming University, Taipei 11221, Taiwan; 3 School of Medicine, Chung Shan Medical University, Taichung 40201, Taiwan; 4 College of Science, Tunghai University, Taichung 407, Taiwan

**Keywords:** brain-derived neurotrophic factor, coronary artery disease, percutaneous coronary intervention, renalase

## Abstract

Circulating brain-derived neurotrophic factor (BDNF) predicts survival rate in patients with coronary artery disease (CAD). We examined the relationship between BDNF and renalase before and after percutaneous coronary intervention (PCI) and the role of renalase in patients with CAD. Serum BDNF and renalase levels were determined using blood samples collected before and after PCI. Incident myocardial infarction, stroke, and mortality were followed up longitudinally. A total of 152 patients completed the assessment. BDNF levels were not significantly changed after PCI compared to baseline levels (24.7 ± 11.0 vs. 23.5 ± 8.3 ng/mL, *p* = 0.175), although renalase levels were significantly reduced (47.5 ± 17.3 vs. 35.9 ± 11.3 ng/mL, *p* < 0.001). BDNF level before PCI was an independent predictor of reduction in renalase (95% confidence interval (CI): −1.371 to −0.319). During a median 4.1 years of follow-up, patients with serum renalase levels of ≥35 ng/mL had a higher risk of myocardial infarction, stroke, and death than those with renalase of <35 ng/mL (hazard ratio = 5.636, 95% CI: 1.444–21.998). In conclusion, our results show that serum BDNF levels before PCI were inversely correlated with the percentage change in renalase levels after PCI. Nevertheless, post-PCI renalase level was a strong predictor for myocardial infarction, stroke, and death.

## 1. Introduction

Coronary artery disease (CAD) is a leading cause of mortality, and CAD-related death has continued to increase over the last decade [1]. Furthermore, CAD is associated with disorders of the central nervous system, including cognitive impairment and depression [2,3,4,5]. Cognitive impairment and depression have been reported to predict cardiovascular mortality in patients with established CAD [6,7]. Hence, psychological assessments are important in patients with CAD. However, objective assessments, including magnetic resonance imaging of the brain, may not fully explain the cognitive decline in patients with CAD [8].

Brain-derived neurotrophic factor (BDNF), a member of the neurotrophin family, is involved in neural development and plays a protective role in central nervous system disorders [9,10]. A low serum BDNF concentration is associated with inflammation and CAD [11,12]. In patients with established CAD, a low serum BDNF concentration is associated with cognitive dysfunction and depressive symptoms [13,14]. Circulating BDNF levels reportedly can predict survival in patients with angina [15]. Percutaneous coronary intervention (PCI) is an important intervention for obstructive coronary artery lesions associated with angina [16,17]. However, depressive symptoms may worsen after PCI [18,19]. To our knowledge, no report has discussed changes in circulating BDNF levels after PCI in patients with CAD.

Renalase produced by the kidney is a flavin-adenine-dinucleotide-dependent amine oxidase that metabolizes catecholamines [20]. The circulating concentration of renalase is reportedly increased in patients with chronic kidney disease (CKD) and might predict mortality in this population [21]. CAD is prevalent in patients with CKD, and the pathogenesis between CAD and CKD is complex [22,23]. Renalase is considered one of the mediators between the kidney and heart and, hence, may serve as a therapeutic target in cardiorenal syndrome [24,25,26]. However, the effect of PCI on circulating renalase levels has not been investigated in patients with CAD.

Since it is possible that BDNF and renalase may serve as mediators or as a bridge between the brain and kidney, the underlying actions of BDNF and renalase may be an important target of further investigation in patients with CAD. We hypothesize that circulating renalase levels decrease after relief of myocardial ischemia via PCI. Therefore, in the present study, we aimed to assess serum renalase levels before and after PCI in CAD patients with angina and examined the relationship of BDNF levels with the change in serum renalase levels. In addition, we also examined the association between post-PCI renalase levels and long-term prognosis.

## 2. Materials and Methods

### 2.1. Subjects

This observational study was conducted in Taichung Veterans General Hospital, and participants were enrolled between April 2009 and March 2015. We included adult patients hospitalized for scheduled coronary angiography due to stable angina, and PCI was performed during the hospitalization period. The exclusion criteria were (1) a history of diabetes or fasting glucose levels ≥126 mg/dL (7 mmol/L) during the hospitalization period; (2) presence of schizophrenia, depression, or bipolar disorders; (3) presence of acute or chronic infectious diseases; (4) presence of severe systemic diseases such as malignancy or immune disorder; and (5) pregnancy. The study complied with the Declaration of Helsinki and was approved by the Institutional Review Board of Taichung Veterans General Hospital. Written consent was obtained before the study procedures were performed.

### 2.2. Methods

The protocol was explained to the subjects on admission, and the candidates were screened for study inclusion only after written consent was provided. After screening, blood samples were collected from the patients prior to angiography. After the fasting glucose levels and PCI results were reviewed, eligible patients were scheduled for an outpatient follow-up appointment, during which blood samples were collected. Overnight fasting blood samples of all enrolled subjects were collected for the measurement of glucose, lipid profile, creatinine, renalase, and BDNF concentrations.

During the follow-up period after PCI, we collected data on the first episodes of myocardial infarction, stroke, and death from the medical records of all enrolled patients. We arranged a phone contact for all enrolled living patients between January 2018 and March 2018. We recorded the first episodes of myocardial infarction, stroke, or death, which were confirmed by patients or their family.

Glucose levels were determined using the oxidase-peroxidase method (Wako Diagnostics, Tokyo, Japan). Creatinine and lipid concentrations were determined using commercial kits (Beckman Coulter, Fullerton, CA, USA). The serum renalase concentration was determined using an enzyme-linked immunosorbent assay (ELISA) kit (Wuhan USCN Business Co., Wuhan, China). The intra- and interassay coefficients of variation (CV) for renalase were 10.0% and 12.0%, respectively. The analytical sensitivity for renalase was 1.31 ng/mL. Serum human BDNF levels were determined using an ELISA kit (R&D Systems, Minneapolis, MN, USA). The intra-assay CV for BDNF was 4.1%, and the interassay CV for BDNF was 9.0%. The sensitivity for BDNF was <0.02 ng/mL. The estimated glomerular filtration rate (eGFR) was calculated based on the Modification of Diet in Renal Disease equation as follows: 186 × (serum creatinine (mg/dL))^−1.154^ × (age (years))^−0.203^ (× 0.742, if female) [27].

The percentage occlusion and location of coronary artery lesions were assessed using the angiography viewing workstation via software for quantitative analysis (Philips Inturis Suite, R2.2, Philips Medical Systems, Eindhoven, The Netherlands).

### 2.3. Statistical Analysis

All continuous data are presented as mean ± standard deviation (SD), and categorical data are presented as absolute numbers (with percentages). The duration between angiography and outpatient visit was presented as median (interquartile range), and the Mann-Whitney test was used to evaluate the differences in follow-up duration between groups. The change in each continuous variable is presented as mean with 95% confidence interval (CI), and a paired *t*-test was used to evaluate the differences within a group prior to and after PCI. The McNemar test was used to evaluate the differences in categorical values prior to and after PCI. An independent sample *t*-test was used to evaluate the statistical differences in continuous variables between two groups. Linear regression analyses were used to assess the relationship between percentage change in serum renalase levels and the baseline associated factors. The correlation between two variables was assessed by calculating Pearson’s correlation coefficient (*r*).

During follow-up, the composite endpoint of myocardial infarction, stroke, and all-cause mortality served as the primary outcome. The significance of univariate analysis for the composite endpoint was determined by the log-rank test using Kaplan-Meier analysis. Cox proportional hazards regression analyses were conducted to determine the hazard ratios of high serum renalase to composite endpoint. Statistical analysis was performed using SPSS version 22.0 software (IBM, New York, NY, USA).

## 3. Results

### 3.1. Assessments before and after PCI

**Figure 1:**
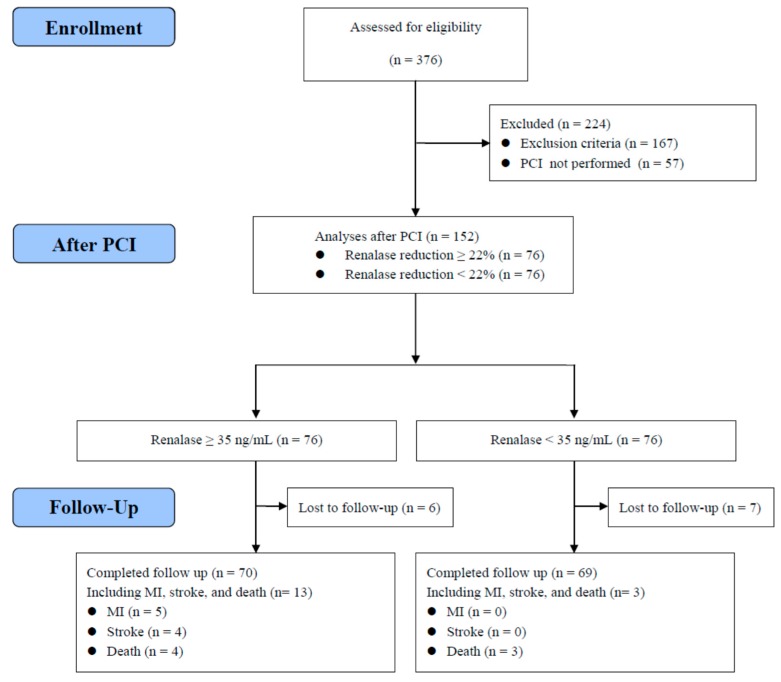
Flow diagram of enrollment of study subjects. Abbreviations: PCI, percutaneous coronary intervention; MI, myocardial infarction.

A total of 152 nondiabetic patients undergoing PCI for CAD during the hospitalization period completed the assessments during the subsequent outpatient visit (Figure 1). The median duration between PCI and the outpatient visit was 9 days (interquartile range, 8–15 days). Table 1 shows the clinical characteristics of the patients before and after PCI. After PCI, the body mass index (BMI) (from 26.8 ± 4.0 to 26.5 ± 3.9 kg/m^2^) and diastolic blood pressure (from 78 ± 14 to 75 ± 10 mmHg) were significantly reduced (*p* < 0.001 and *p* = 0.005, respectively), and high-density lipoprotein (HDL) cholesterol levels were significantly increased (from 1.0 ± 0.3 to 1.2 ± 0.2 mmol/L, *p* < 0.001). After PCI, the proportions of subjects using angiotensin-converting enzyme (ACE) inhibitor/angiotensin II receptor blocker (ARB), β-blocker, antiplatelet agents, and statins were significantly increased. It is notable that the serum BDNF levels were not significantly different after PCI (from 24.7 ± 11.0 to 23.5 ± 8.3 ng/mL, *p* = 0.175), whereas the eGFR (from 84.3 ± 22.5 to 77.0 ± 20.4 mL/min/1.73 m^2^) and serum renalase levels (from 47.5 ± 17.3 to 35.9 ± 11.3 ng/mL) were significantly decreased after PCI (*p* < 0.001 for both). Although the BMI, diastolic blood pressure, HDL cholesterol levels, and eGFR changed significantly after PCI, these alterations were not significantly correlated with the change in the serum renalase levels after PCI (Table 2).

**Figure 2:**
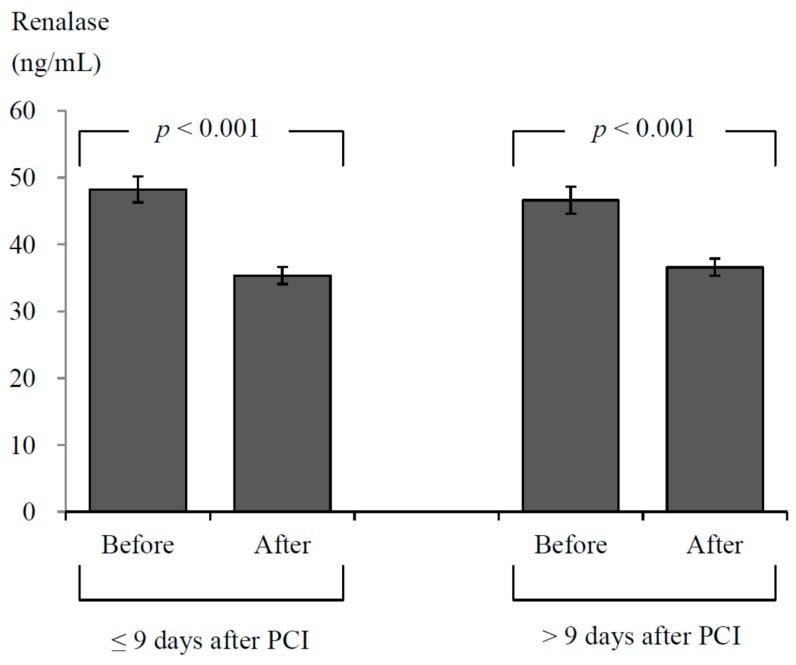
Serum renalase concentrations before and after percutaneous coronary intervention (PCI) in patients who underwent outpatient renalase assessment within nine days of PCI (*n* = 87) and >9 days after PCI (*n* = 65). Mean and standard error of serum renalase levels are shown.

To assess the effects of follow-up duration on change in serum renalase levels after PCI, all patients were divided into two groups based on the median duration between the PCI and outpatient visit. The change in serum renalase levels was significantly decreased after PCI in patients who underwent outpatient assessment within nine days after PCI (from 48.2 ± 18.1 to 35.3 ± 12.0 ng/mL, *p* < 0.001) as well as those who underwent outpatient assessment more than nine days after PCI (from 46.6 ± 16.3 to 36.6 ± 10.3 ng/mL, *p* < 0.001). The change in serum renalase levels did not significantly differ between patients followed within nine days of PCI and those followed more than nine days after PCI (−12.9 ± 17.8 vs. −10.0 ± 14.9 ng/mL, *p* = 0.292; Figure 2).

**Figure 3:**
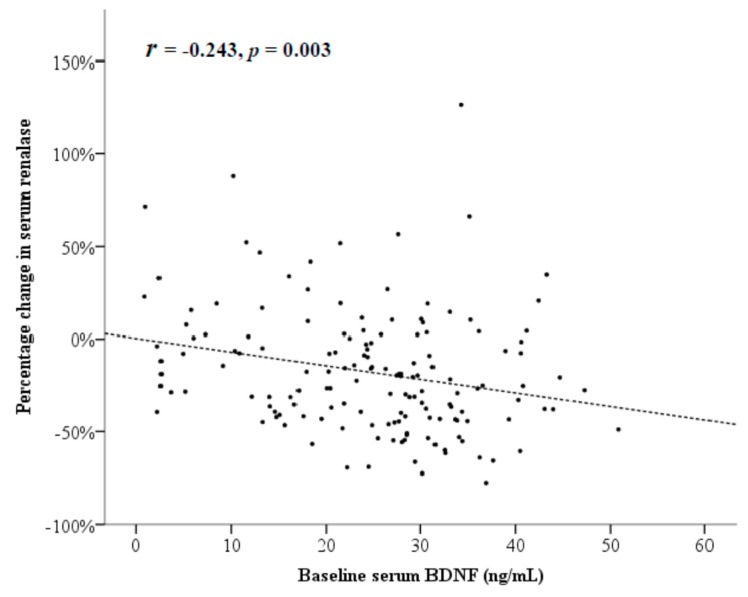
A significant correlation exists between the percentage changes in serum renalase and baseline serum brain-derived neurotrophic factor (BDNF) levels; correlation coefficient (*r*) = −0.243, *p* = 0.003.

To assess the effects of the associated factors at baseline on the change in serum renalase levels after PCI, all patients were assigned to either the renalase reduction group or reference group, according to the median percentage change in serum renalase levels (22%). The median duration between PCI and outpatient visit was not significantly different between the renalase reduction group and the reference group (*p* = 0.615). Higher baseline serum BDNF levels were observed in patients with serum renalase reduction ≥22%, as compared to the other patients (27.0 ± 10.1 vs. 22.4 ± 11.3 ng/mL, *p* = 0.009; Table 3). Figure 3 shows that the percentage change in serum renalase levels was inversely correlated with the baseline serum BDNF levels (*r* = −0.243, *p* = 0.003).

On univariate regression analysis, the use of anti-hypertensive drugs, antiplatelet drugs, or statins was not associated with the percentage reduction in serum renalase levels. Only baseline serum BDNF levels were significantly associated with percentage reduction in serum renalase levels (linear regression coefficient = −0.736, 95% CI: −1.209 to −0.263, *p* = 0.003). On multivariate linear regression analysis, baseline serum BDNF was still independently associated with percentage reduction in serum renalase (linear regression coefficient = −0.845, 95% CI: −1.371 to −0.319, *p* = 0.002) after adjusting for age, gender, smoking, BMI, systolic blood pressure, eGFR, fasting glucose levels, and lipid levels (Table 4).

### 3.2. Assessments for Longitudicnal Follow-Up

**Figure 4:**
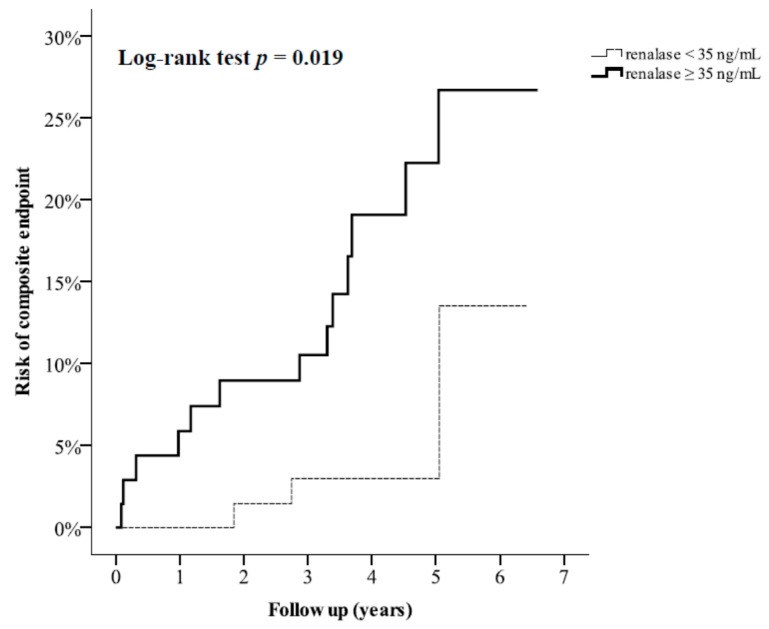
Kaplan-Meier curves showing risk of composite endpoint (incident myocardial infarction, stroke, or death) categorized according to median serum renalase (35 ng/mL) after percutaneous coronary intervention (PCI).

The median follow-up duration was 4.1 years. The data related to the composite endpoint were collected via phone contact for 139 patients, identifying five patients with a first event of myocardial infarction, four patients with a first event of stroke, and four patients who died without known myocardial infarction and stroke during follow-up (Figure 1). To assess the effect of post-PCI serum renalase on composite endpoint, we divided all patients into two equal-sized categories based on serum renalase levels (median of 35 ng/mL). Figure 4 shows that composite endpoint was significantly different between higher renalase and lower renalase groups, as demonstrated by Kaplan-Meier analysis (log-rank test *p* = 0.019). Using multivariate Cox regression analysis, we found that patients with serum renalase ≥35 ng/mL had higher risk (hazard ratio = 5.636, 95% CI: 1.444–21.998; *p* = 0.013) for the composite endpoint compared to patients with serum renalase <35 ng/mL after adjustment for age, gender, statin treatment, number of coronary arteries with significant narrowing, total cholesterol, and eGFR (Table 5).

## 4. Discussion

In the present study, our main findings are that serum renalase levels were significantly reduced after PCI in patients with established CAD, although the serum BDNF levels did not significantly change. In addition, the pre-PCI serum BDNF levels were significantly associated with a reduction in serum renalase levels after PCI. In line with our findings before and after PCI, Wybraniec et al. [28] reported that urinary renalase levels were significantly decreased after PCI in patients with CAD.

Furthermore, we also observed that a low post-PCI serum renalase level could predict a lower risk for the composite endpoint including a first episode of myocardial infarction, stroke, and all-cause mortality. In line with our longitudinal follow-up findings for patients with established CAD, high baseline serum renalase levels were found to predict a high risk of all-cause mortality in patients with CKD in a prospective observational study [21], although a low plasma renalase concentration was observed in patients with CAD in a cross-sectional study [29].

Renalase was reported to have a protective effect on the kidney by preventing fibrosis following acute renal injury in rats after unilateral ureteral obstruction [30] and by reducing inflammation following ischemic acute renal injury in mice [31]. Zhao et al. [32] found that pretreatment with renalase prevented contrast-induced kidney injury in rats. However, high circulating renalase levels were also found to be associated with a decline in renal function in renal transplant recipients [33]. In the present study, eGFR was significantly decreased after PCI. However, the reduction in eGFR was not significantly associated with a change in the serum renalase levels.

In addition to being released from the kidney, renalase is also reportedly synthesized in the brain and plays a role in regulating monoamine neurotransmitter activity [34]. As BDNF can potentially increase dopamine levels in the central nervous system [35], it is reasonable to hypothesize that BDNF is involved in the regulation of renalase production, and renalase inhibition may therefore be associated with a change in monoamine oxidase activities and catecholamine concentrations in both the brain and peripheral circulation of mice [36]. In the present study, serum BDNF levels before PCI were significantly correlated with the reduction in serum renalase levels after PCI. Our findings also demonstrated the potential inhibitory effect of BDNF on renalase in patients with established CAD.

It has been reported that a low serum BDNF level could predict the occurrence of cardiovascular events [15,37]. Another important finding of the present study is that the serum BDNF levels did not significantly change before and after PCI in patients with established CAD. Therefore, serum BDNF could serve as a relatively stable marker for cardiovascular risk before and after PCI. We observed a reduction in serum renalase levels after PCI in patients with established CAD. The association between mortality risk and serum renalase levels has previously only been reported in patients with CKD [21], and our longitudinal follow-up provides evidence for a relationship between serum renalase levels and risk of myocardial infarction, stroke, and all-cause mortality.

Nevertheless, the present study has certain limitations. First, we did not perform cognitive assessments. Serum BDNF has previously been reported to be significantly correlated with the mini-mental state examination score [13]. Although cognitive decline is observed after coronary angiography [38], we did not observe a significant change in serum BDNF levels in the present study. Second, we did not assess the mechanism underlying the inhibitory effect of serum BDNF on renalase after PCI, and we did not assess the source from which renalase is released to the circulation after PCI. Third, different coronary BDNF expression had been reported between stable and unstable angina [39]. The present study findings could not be applied in patients with unstable angina or acute myocardial infarction because we only enrolled patients in stable condition for scheduled PCI. Fourth, we did not enroll healthy subjects as controls for comparing the serum renalase levels to those with established CAD. Finally, we could not control several potential biases for prognosis, such as type of stents during PCI or use of medications during follow-up, in this observational study.

## 5. Conclusions

In conclusion, serum renalase levels were significantly decreased after PCI, and the reduction in serum renalase was significantly correlated with the serum BDNF concentrations before PCI in patients with established CAD. Furthermore, a low serum renalase level could predict a lower risk for composite endpoint of myocardial infarction, stroke, and death. Further investigations of the mechanism between serum renalase and all-cause or cardiovascular mortality risk are needed in patients with established CAD.

## Figures and Tables

**Table 1:** Clinical data of patients (*n* = 152) before and after percutaneous coronary intervention (PCI).

	Before *	After *	Change ^#^	*p* ^†^	
Age (years)	60 ± 12				
Male, *n* (%)	136 (89.5%)				
Current smoker, *n* (%)	47 (30.9%)				
BMI (kg/m^2^)	26.8 ± 4.0	26.5 ± 3.9	−0.3	(−0.5, −0.1)	<0.001
Systolic BP (mmHg)	128 ± 19	129 ± 18	0.2	(−2.9, 3.4)	0.877
Diastolic BP (mmHg)	78 ± 14	75 ± 10	−3.2	(−5.4, −1.0)	0.005
Fasting glucose (mmol/L)	5.6 ± 1.1	5.4 ± 0.9	−0.2	(−0.3, 0.0)	0.108
Total cholesterol (mmol/L)	4.1 ± 1.1	4.1 ± 0.9	−0.1	(−8.7, 4.3)	0.506
HDL cholesterol (mmol/L)	1.0 ± 0.3	1.2 ± 0.2	0.2	(5.0, 7.5)	<0.001
Triglyceride (mmol/L)	1.4 ± 0.9	1.5 ± 0.8	0.04	(−6.5, 13.3)	0.498
eGFR (mL/min/1.73 m^2^)	84.3 ± 22.5	77.0 ± 20.4	−7.3	(−9.7, −4.9)	<0.001
BDNF (ng/mL)	24.7 ± 11.0	23.5 ± 8.3	−1.1	(−2.8, 0.5)	0.175
Renalase (ng/mL)	47.5 ± 17.3	35.9 ± 11.3	−11.7	(−14.3, −9.0)	<0.001
Antihypertensive agent use					
ACE inhibitor or ARB, *n* (%)	74 (48.7%)	108 (71.1%)	34.0	(45.9%)	<0.001
α-blocker, *n* (%)	12 (7.9%)	6 (3.9%)	−6.0	(−50.0%)	0.070
β-blocker, *n* (%)	31 (20.4%)	46 (30.3%)	15.0	(48.4%)	0.009
Calcium channel blocker, *n* (%)	73 (48.0%)	73 (48.0%)	0.0	(0.0%)	0.999
Diuretics, *n* (%)	23 (15.1%)	25 (16.4%)	2.0	(8.7%)	0.754
Antiplatelet agent, *n* (%)	125 (82.2%)	148 (97.4%)	23.0	(18.4%)	<0.001
Statins, *n* (%)	79 (52.0%)	115 (75.7%)	36.0	(45.6%)	<0.001

ACE = angiotensin-converting enzyme, ARB = angiotensin II receptor blocker, BDNF = brain-derived neurotrophic factor, BMI = body mass index, BP = blood pressure, eGFR = estimated glomerular filtration rate, HDL = high-density lipoprotein. * continuous data are presented as mean ± standard deviation, and categorical data are presented as integer values (percentages) ^#^ continuous data are presented as mean (95% confidence interval), and categorical data are presented as integer values (percentages) ^†^ denotes *p* values for differences before and after PCI.

**Table 2:** The correlation between the percentage change in serum renalase levels and changes in cardiovascular risk factors significantly altered after percutaneous coronary intervention (PCI).

	*r*	*p*
ΔBMI (kg/m^2^)	−0.103	0.209
ΔDiastolic BP (mmHg)	0.099	0.225
ΔHDL cholesterol (mmol/L)	0.110	0.176
ΔGFR (mL/min/1.73 m^2^)	−0.011	0.896

Δ = (variable after PCI − variable before PCI)/variable before PCI), BMI = body mass index, BP = blood pressure, eGFR = estimated glomerular filtration rate, HDL = high-density lipoprotein, PCI = percutaneous coronary intervention.

**Table 3:** Clinical data before and after percutaneous coronary intervention (PCI) among patients categorized based on the median percentage change in renalase levels (22%).

	Renalase Reduction (*n* = 76)	Reference (*n* = 76)	*p* *	*p* ^#^
Demographic characteristics				
Age (years)	60 ± 11	60 ± 12	0.812	
Male, *n* (%)	68 (89.5%)	68 (89.5%)	1.000	
Current smoker, *n* (%)	20 (26.3%)	27 (35.5%)	0.292	
Anthropometric data				
BMI (kg/m^2^)				
Before (mean ± SD)	27.1 ± 3.8	26.6 ± 4.2	0.530	
After (mean ± SD)	26.8 ± 3.6	26.2 ± 4.1		
Change (mean (95% CI))	−0.2 (−0.5, 0.1)	−0.4 (−0.6, 0.2)		0.353
Systolic BP (mmHg)				
Before (mean ± SD)	128 ± 16	129 ± 21	0.745	
After (mean ± SD)	128 ± 17	129 ± 19		
Change (mean (95% CI))	1 (−3, 5)	0 (−5, 5)		0.774
Diastolic BP (mmHg)				
Before (mean ± SD)	79 ± 13	76 ± 15	0.138	
After (mean ± SD)	75 ± 10	74 ± 10		
Change (mean (95% CI))	−4 (−7, −1)	−2 (−5, 1)		0.286
Biochemistry data				
Fasting glucose (mmol/L)				
Before (mean ± SD)	5.6 ± 1.3	5.6 ± 1.0	0.969	
After (mean ± SD)	5.4 ± 0.8	5.4 ± 1.0		
Change (mean (95% CI))	−0.1 (−0.4, 0.1)	−0.2 (−0.5, 0.1)		0.787
Total cholesterol (mmol/L)				
Before (mean ± SD)	4.1 ± 1.1	4.1 ± 1.1	0.789	
After (mean ± SD)	4.0 ± 0.9	4.1 ± 0.9		
Change (mean (95% CI))	−0.1 (−0.4, 0.1)	0.0 (−0.2, 0.3)		0.283
HDL cholesterol (mmol/L)				
Before (mean ± SD)	1.0 ± 0.3	1.0 ± 0.3	0.789	
After (mean ± SD)	1.1 ± 0.2	1.2 ± 0.2		
Change (mean (95% CI))	0.2 (0.1, 0.2)	0.2 (0.1, 0.2)		0.573
Triglyceride (mmol/L)				
Before (mean ± SD)	1.5 ± 1.1	1.3 ± 0.8	0.157	
After (mean ± SD)	1.5 ± 0.9	1.4 ± 0.8		
Change (mean (95% CI))	0.0 (−0.2, 0.2)	0.1 (0.0, 0.2)		0.305
eGFR (mL/min/1.73m^2^)				
Before (mean ± SD)	87.0 ± 20.3	81.6 ± 24.3	0.140	
After (mean ± SD)	78.8 ± 17.0	75.2 ± 23.4		
Change (mean (95% CI))	−8.2 (−11.8, −4.5)	−6.4 (−9.5, 3.4)		0.467
BDNF (ng/mL)				
Before (mean ± SD)	27.0 ± 10.1	22.4 ± 11.3	0.009	
After (mean ± SD)	22.7 ± 8.1	24.3 ± 8.5		
Change (mean (95% CI))	−4.2 (−6.3, −2.2)	2.0 (−0.5, 4.5)		<0.001

BDNF = brain-derived neurotrophic factor, BMI = body mass index, BP = blood pressure, CI = confidence interval, eGFR = estimated glomerular filtration rate, HDL = high-density lipoprotein, SD = standard deviation * denotes *p*-values for differences in baseline data between the two groups ^#^ denotes *p*-values for differences in the change before and after PCI between the two groups.

**Table 4:** Effect of baseline risk factors on the percentage change serum renalase levels after percutaneous coronary intervention (PCI).

	Univariate Linear Regression Analysis			Multivariate Linear Regression Analyses					
	B ^#^	95% CI	*p*	B ^#^	95% CI	*p*	B ^#^	95% CI	*p*
Age (every 10 years)	0.511	(−4.093, 5.115)	0.827	−0.356	(−5.106, 4.395)	0.883	−0.323	(−5.756, 5.110)	0.907
Male (yes/no)	−5.977	(−23.303, 11.350)	0.497	−11.310	(−28.982, 6.363)	0.208	−11.842	(−30.897, 7.212)	0.221
Current smoker (yes/no)	2.284	(−9.233, 13.801)	0.696	8.144	(−3.881, 20.168)	0.183	8.868	(−3.757, 21.494)	0.167
BMI (kg/m^2^)	−0.259	(−1.596, 1.079)	0.703				−0.059	(−1.631, 1.514)	0.941
Systolic BP (mmHg)	0.121	(−0.165, 0.407)	0.404				0.117	(−0.185, 0.418)	0.446
Diastolic BP (mmHg)	−0.210	(−0.598, 0.178)	0.287						
eGFR (mL/min/1.73m^2^)	−0.068	(−0.305, 0.170)	0.574				0.041	(−0.220, 0.302)	0.758
Fasting glucose (mmol/L)	2.025	(−2.627, 6.678)	0.391				2.128	(−2.701, 6.957)	0.385
Total cholesterol (mmol/L)	−0.087	(−5.117, 4.943)	0.973				0.430	(−5.683, 6.543)	0.890
Triglyceride (mmol/L)	−2.091	(−7.777, 3.595)	0.469				2.375	(−23.369, 28.119)	0.856
HDL cholesterol (mmol/L)	1.600	(−19.207, 22.406)	0.879				−2.728	(−9.590, 4.134)	0.433
BDNF (ng/mL)	−0.736	(−1.209, −0.263)	0.003	−0.851	(−1.345, −0.357)	0.001	−0.845	(−1.371, −0.319)	0.002
ACE inhibitor or ARB used	3.040	(−7.604, 13.683)	0.573						
α-blocker used	−5.087	(−24.819, 14.645)	0.611						
β-blocker used	3.429	(−9.776, 16.635)	0.609						
Calcium channel blocker used	−2.190	(−12.843, 8.464)	0.685						
Diuretics used	2.626	(−12.229, 17.481)	0.727						
Antiplatelet agent used	−6.749	(−20.875, 7.377)	0.347						
Statins used	1.927	(−8.728, 12.582)	0.721						

^#^ B = linear regression coefficient ACE = angiotensin-converting enzyme, ARB = angiotensin II receptor blocker, BDNF = brain-derived neurotrophic factor, BMI = body mass index, BP = blood pressure, CI = confidence interval, eGFR = estimated glomerular filtration rate, HDL = high-density lipoprotein.

**Table 5:** Effect of serum renalase levels after percutaneous coronary intervention (PCI) on composite endpoint (incident myocardial infarction, stroke, or all-cause mortality).

	Univariate Model			Multivariate Model					
	Crude			Model 1			Model 2		
	HR	95% CI	*p*	HR	95% CI	*p*	HR	95% CI	*p*
Serum renalase ≥ 35 ng/mL (yes/no)	4.031	(1.141, 14.236)	0.030	3.674	(1.041, 12.972)	0.043	5.636	(1.444, 21.998)	0.013
Age ≥ 60 years (yes/no)	3.927	(1.262, 12.218)	0.018	3.720	(1.168, 11.851)	0.026	5.284	(1.556, 17.946)	0.008
Gender (male/female)	0.755	(0.171, 3.331)	0.711	1.163	(0.258, 5.253)	0.844	1.112	(0.200, 6.192)	0.903
Using statins after PCI (yes/no)	0.910	(0.290, 2.856)	0.872				1.223	(0.334, 4.486)	0.761
Multiple coronary artery disease (yes/no)	1.263	(0.457, 3.493)	0.653				1.406	(0.490, 4.034)	0.526
Total cholesterol (increase in every 1 mmol/L)	1.048	(0.626, 1.754)	0.860				1.157	(0.627, 2.135)	0.641
eGFR (increase in every 15 mL/min/1.73m^2^)	0.982	(0.669, 1.442)	0.928				1.470	(0.967, 2.234)	0.071

CI = confidence interval, eGFR = estimated glomerular filtration rate, HR = hazard ratio, PCI = percutaneous coronary intervention.
